# Identification and development of Tetra-ARMS PCR-based screening test for a genetic variant of OLA1 (Tyr254Cys) in the human failing heart

**DOI:** 10.1371/journal.pone.0293105

**Published:** 2024-06-18

**Authors:** Praveen K. Dubey, Shubham Dubey, Sarojini Singh, Purnima Devaki Bhat, Steven Pogwizd, Prasanna Krishnamurthy

**Affiliations:** 1 Department of Biomedical Engineering, Schools of Medicine and Engineering, University of Alabama at Birmingham, Birmingham, Alabama, United States of America; 2 Comprehensive Cardiovascular Center, Department of Medicine, University of Alabama at Birmingham, Birmingham, Alabama, United States of America; Kyoto University Graduate School of Medicine Faculty of Medicine: Kyoto Daigaku Daigakuin Igaku Kenkyuka Igakubu, JAPAN

## Abstract

Obg-like ATPase 1 (OLA1) protein has GTP and ATP hydrolyzing activities and is important for cellular growth and survival. The human *OLA1* gene maps to chromosome 2 (locus 2q31.1), near *Titin* (*TTN*), which is associated with familial dilated cardiomyopathy (DCM). In this study, we found that expression of OLA1 was significantly downregulated in failing human heart tissue (HF) compared to non-failing hearts (NF). Using the Sanger sequencing method, we characterized the human *OLA1* gene and screened for mutations in the *OLA1* gene in patients with failing and non-failing hearts. Among failing and non-failing heart patients, we found 15 different mutations in the *OLA1* gene, including two transversions, one substitution, one deletion, and eleven transitions. All mutations were intronic except for a non-synonymous 5144A>G, resulting in 254Tyr>Cys in exon 8 of the *OLA1* gene. Furthermore, haplotype analysis of these mutations revealed that these single nucleotide polymorphisms (SNPs) are linked to each other, resulting in disease-specific haplotypes. Additionally, to screen the 254Tyr>Cys point mutation, we developed a cost-effective, rapid genetic screening PCR test that can differentiate between homozygous (AA and GG) and heterozygous (A/G) genotypes. Our results demonstrate that this PCR test can effectively screen for OLA1 mutation-associated cardiomyopathy in human patients using easily accessible cells or tissues, such as blood cells. These findings have important implications for the diagnosis and treatment of cardiomyopathy.

## Introduction

Obg-like ATPase 1 (OLA1) is a member of the GTPase family of proteins, exhibiting both GTPase and ATPase activities. It is highly expressed in cancer cells and is associated with poor survival [1, 2]. In cancer cells, increased expression of OLA1 inhibits apoptosis by interacting with breast cancer-associated gene 1 (BRCA1) and BRCA1-associated RING domain protein (BARD1) [3]. OLA1 also interacts with eIF2α to form a ternary complex for regulating protein translation and cell proliferation [4]. Recent studies have shown that OLA1 interacts with Hsp70 and stabilizes mitochondrial superoxide dismutase 2 (SOD2) in mouse pulmonary smooth muscle cells (SMCs). Loss of OLA1 caused SOD2 deficiency, resulting in the increased expression of the X-linked inhibitor of apoptosis (*XIAP*) gene and increased proliferation of pulmonary SMCs [5, 6].

A recent proteomics study in human hypertrophic cardiomyopathy (HCM) patients showed that the expression of several genes, including *OLA1*, associated with a failing heart, was downregulated [7]. Our previous study in mice also found that treating mice with angiotensin II (AngII) altered the expression of OLA1, resulting in the phosphorylation of the GSK-3β/β-catenin pathway [8]. This pathway plays a crucial role in the development of cardiac hypertrophy [8]. Further study in mice has shown that deletion of the *OLA1* gene led to an enlargement of the heart [4], indicating the potential impact of OLA1 on cardiac structure and, consequently function.

Although mutations in more than 40 genes are known to cause DCM in humans [9, 10], it is not known if mutations in the *OLA1* gene have any direct role in human heart failure. In this study, we sought to screen for mutations in the *OLA1* gene among failing and non-failing patients and investigate their association with heart failure. Additionally, we also developed a cost-effective and rapid PCR-based genetic screening test using DNA samples to identify both homozygous and heterozygous mutations in the *OLA1* gene. This test should help in the further investigation of the role of OLA1 in heart failure and develop therapeutic approaches in such cases.

## Materials and methods

### Patient samples

We obtained anonymized heart tissue samples from the tissue repository facility at the University of Alabama at Birmingham (UAB). The samples included non-failing (n = 5; NF1-5) and failing hearts (n = 5; HF6-10). The classification of heart tissues as failing or non-failing was based on clinical diagnoses made prior to sample collection. Patients’ identities were not known to the research group, ensuring complete anonymity. The UAB Institutional Review Board approved the study protocols.

### Collection of mice tissue samples

Mice tissue samples were collected from wild-type C57BL/6J mice (10-12-week-old) purchased from the Jackson Laboratory, Bar Harbor, ME. All protocols were approved by the UAB Institutional Animal Care and Use Committee (IACUC).

### RNA isolation and cDNA synthesis

Total RNA was isolated from ~50 mg of failing and non-failing human heart tissues using the TRIzol method (Invitrogen) and purified with the Qiagen RNA extraction kit (cat# 74106, Qiagen). RNA quality and quantity were assessed with NanoDrop (Thermo Fisher Scientific), and 1 μg of RNA from each sample was reverse transcribed using the RevertAid First Strand cDNA Synthesis Kit (cat# K1691, Thermo Fisher Scientific). Specific primers (S1 Table) were used for performing qPCR analyses on a QuantStudio 3 system (Applied Biosystems, Thermo Fisher Scientific) using the PowerUp^™^ SYBR^™^ Green Master Mix (cat# A25778, Thermo Fisher Scientific). Target gene expression was normalized to a housekeeping gene (GAPDH) and presented as fold change.

### DNA isolation

Total DNA from failing and non-failing human heart tissues (~5 mg) was isolated using the Qiagen DNAeasy kit (cat# 69504, Qiagen) according to the manufacturer’s instructions. The quality and quantity of the isolated DNA were measured using NanoDrop (Thermo Fisher Scientific).

### Primer design and PCR amplification

To screen polymorphisms in the *OLA1* gene, nine sets of primers (S1 Table) were designed using the Primer Blast server of NCBI. The available human *OLA1* gene sequences from the ensemble genome browser (Transcript: ENST00000284719.8) were used in designing primers. PCR for *OLA1* was performed to cover all the exons along with partial introns using the genomic DNA from failing and non-failing heart tissues. Before sequencing, all PCR products were optimized for mutant-specific amplification. Details of the PCR primers, along with their annealing temperatures and size, are given in S1 Table. For each PCR reaction, a total volume of 20 μL containing 20–50 ng genomic DNA, 10 pmoL of each primer, and 2× PCR Master Mix (cat# PRM7505, Promega) was used. After an initial denaturation step (95°C for 3 min), samples were subjected to 32 cycles of PCR consisting of 94°C for 30 sec, primer-specific annealing temperature (see S1 Table) for 30 sec, and 72°C for 90 sec, followed by a final extension of 7 min at 72°C. Amplified products were run on a 1.5% agarose gel, and images were captured using Gel Doc^™^ EZ System (Bio-Rad).

### DNA sequencing and analyses

Before Sanger sequencing, the amplified PCR products were enzymatically cleaned up using ExoSAP-IT^™^ PCR Product Cleanup Reagent (cat# 75001.200UL, Thermo Fisher Scientific). Subsequently, the cleaned PCR products were sequenced using the Sanger method at the UAB core facility. Each PCR product was sequenced with its respective PCR primer using the fluorescent dideoxy terminator (ABI Prism BigDye Terminator v3.1 Cycle Sequencing kit). The sequencing reactions were purified using the Applied Biosystems BigDye Terminator purification system per the manufacturer’s protocols. After purification, sequencing reactions were run on a 3730xl DNA Analyzer (Applied Biosystems). The sequence of each DNA sample was manually checked using the Chromas Lite program (http://www.technelysium.com.au/chromas_lite.html) and further subjected to multiple alignments to identify nucleotide variations, using the MegAlign program of the Lasergene software. Finally, different fragments of the *OLA1* gene sequences were assembled using the SeqMan program of Lasergene and then submitted to the NCBI GenBank.

### Genetic screening test for the *OLA1* point mutation (254 Tyr>Cys)

For genotyping of the non-synonymous SNP 5144A>G, which results in the amino acid change 254Tyr>Cys in Exon 8 of the *OLA1* gene, a tetra-primers ARMS-PCR-based screening protocol was developed using four different sets of primers (S1 Table) as previously described [11–13]. The primers were designed from the human *OLA1* sequence (accession no. ON073791.1) using a web-based software available at http://cedar.genetics.soton.ac.uk/public_html/primer1.html and synthesized through Integrated DNA Technologies (IDT). Nucleotide-specific amplification protocol was standardized and validated by S anger sequencing. PCR was performed on isolated genomic DNA samples in a 20 μL reaction volume containing approximately 25–50 ng of the template DNA, 0.5 μL of 10 pmoL of each inner primer, 0.1 μL of 10 pmoL of each outer primer, and 2× PCR Master Mix (cat# PRM7505, Promega). PCR-amplification was carried out using the following conditions: 95°C for 3 min, followed by 32 cycles of 94°C for 30 sec, annealing at 58°C for 30 sec, and 72°C for 1 min, followed by a final extension at 72°C for 5 min. The amplified PCR products were visualized on ethidium bromide-stained 2% ultra-pure agarose gel, and genotypes were recorded.

### Protein extraction and western blot analysis

Protein lysates from failing and non-failing human hearts and different tissues from mice were prepared using the RIPA Cell Lysis Buffer (cat# J63324, Alfa Aesar) supplemented with Halt^™^ protease inhibitor (cat# 87786, Thermo Fisher Scientific) and phosphatase inhibitor (cat# P5726, Millipore Sigma) cocktails according to the manufacturer’s instructions. Equal amounts of the proteins were loaded on Any kD^™^ Mini-PROTEAN TGX stain-free protein gels (cat# 4569033, Bio-Rad Laboratories) and transferred to polyvinylidene difluoride (PVDF) membranes using Trans-Blot Turbo transfer system (Bio-Rad). The blots were quickly washed with water and blocked with 5% non-fat milk for one hour at room temperature. The blots were incubated with primary antibodies against OLA1 (cat# 16371-1-AP, Proteintech) and β-Tubulin (cat# 10094-1-AP, Proteintech) overnight at 4°C. After washing, blots were incubated with secondary HRP-conjugated antibodies against mice (cat# SA00001-1, Proteintech) and rabbits (cat# SA00001-2, Proteintech) at room temperature for 1 hour. Images were acquired on a ChemiDoc^™^ Touch Imaging System (Bio-Rad) using an enhanced chemiluminescence (Pierce) detection system.

### Cell culture and protein fractionation

Human ventricular cardiomyocyte cell line (AC16; cat# SCC109, Sigma-Aldrich) was cultured in DMEM/F12 (Cat# D6434, Sigma-Aldrich) supplemented with 2 mM L-Glutamine (cat# TMS-002-C, EMD Millipore), 12.5% fetal bovine serum (FBS; cat# ES-009-B, EMD Millipore) and 1% penicillin-streptomycin solution (cat# 15140122, Thermo Fisher Scientific) in a humidified incubator at 37°C with 5% CO_2_. Cells were seeded in a 10 cm dish for cytoplasmic and nuclear fractionation. NE-PER^™^ Nuclear and Cytoplasmic Extraction Kit (cat# 78835, Thermo Fisher Scientific) was used for cytoplasmic and nuclear fractionation. Protease and phosphatase inhibitors were added before use. Fractionated proteins were separated on an SDS-PAGE, the proteins were transferred to the PVDF membrane, and western analysis was done as above with antibodies against OLA1, GAPDH (cytoplasmic marker), and Lamin B1 (Nuclear marker).

### Statistical analyses

The data were analyzed using a two‐tailed unpaired t-test for comparison of two groups using GraphPad. All the values were presented as mean ± SEM. The probability(p) values of <0.05 were considered statistically significant.

## Results

### Expression of OLA1 was downregulated in failing human heart

**Fig 1:**
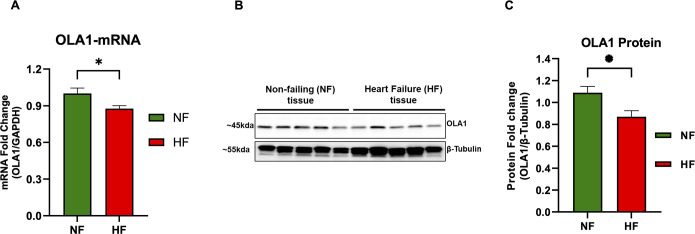
Expression of OLA1 in failing (HF) and non-failing human heart (NF). (A) mRNA quantification of the *OLA1* gene using quantitative real-time PCR in a subset of the failing heart tissue (n = 5; HF) and non-failing heart tissue (n = 5; NF). Glyceraldehyde 3-phosphate dehydrogenase (GAPDH) served as internal control for normalization. The data shown as relative fold change. (B) A representative immunoblot showing OLA1 protein expression in human heart with severe left ventricular dysfunction (non-ischemic, heart failure, n = 5, HF) and non-failing control subjects (n = 5). (C) The densitometric quantification of immunoblots. The data are presented as fold change and expressed as mean ± SEM. Data were analyzed using a two-tailed unpaired student *t*-test, with significance set at *p < 0.05.

OLA1 has been shown to play a pivotal role in the progression of cancer and heart disease [2, 7, 8, 14]. In this study, we assessed the expression of OLA1 (mRNA and protein) in human failing (HF) and non-failing (NF) heart tissues. Interestingly, our qPCR analysis indicated a significant downregulation of *OLA1* mRNA in failing, compared to non-failing human heart tissue (Fig 1A). Furthermore, western blot analysis of proteins from the same tissues revealed a concurrent reduction in the OLA1 protein expression in failing relative to non-failing hearts (Fig 1B and 1C). These findings collectively suggest a decreased expression of OLA1 in the failing heart compared to the non-failing heart. This decreased expression of *OLA1* in the failing heart is seen both at the mRNA and protein levels (Fig 1A–1C).

To determine tissue-specificity in the expression of OLA1, we performed Western blot analysis of various mouse tissues, including lungs, kidneys, heart, liver, spleen, and skeletal muscle. This immunoblotting analysis revealed that the OLA1 protein is ubiquitously expressed in all tissues (S1A and S1B Fig). Furthermore, subcellular fractionation of cytoplasmic and nuclear proteins from immortalized human ventricular cardiomyocytes (AC16 cells) demonstrated that OLA1 primarily localizes in the cytoplasm; however, low levels of OLA1 were also observed in the nucleus (S1C Fig). Although OLA1 is predominantly localized in the cytoplasm, this result shows that it can translocate to the nucleus. Its post-translational modification, such as phosphorylation (Ser232/Tyr236), may mediate nuclear shuttling [15].

### Characterization of the human *OLA1* gene

To comprehensively characterize the human *OLA1* gene at both mRNA and genomic levels, we amplified mRNA and genomic sequences of the *OLA1* gene using RNA and DNA extracted from human heart tissue. For mRNA characterization, we amplified the entire Open Reading Frame (ORF) of the *OLA1* mRNA and cloned it into the TOPO TA cloning vector (cat# 450071). Using Sanger sequencing, various fragments of the *OLA1* sequence were assembled and subjected to NCBI Blast analysis to assess their homology. Upon confirmation of the sequence accuracy for the human *OLA1* gene, the *OLA1* mRNA was translated into amino acids and deposited in the NCBI database under the accession number ON073790.1.

**Fig 2:**
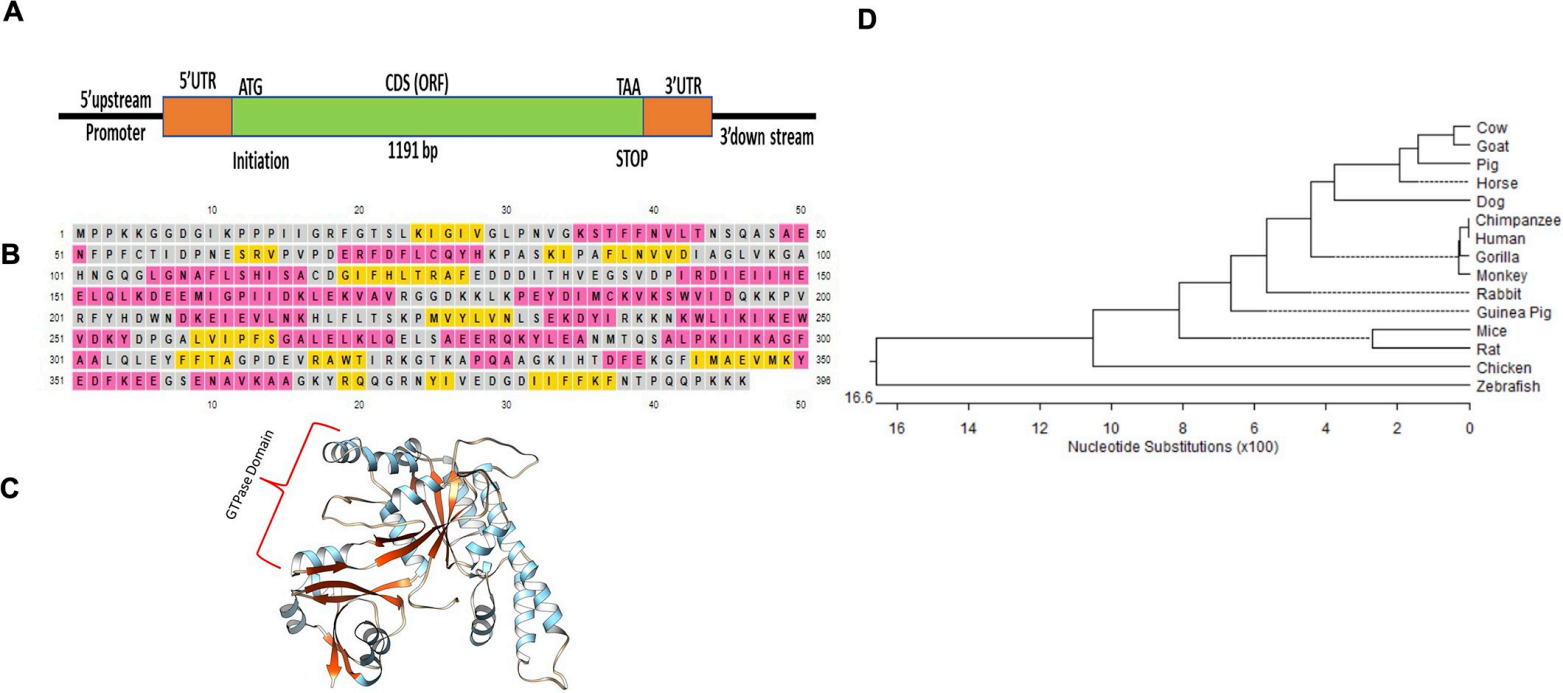
The characterization of the human *OLA1* gene. Panel (A) shows the amplified *OLA1* mRNA. The coding sequence (CDS) is shown in green (1191bp), along with the 5’-UTR and 3’-UTR sequence. Panel (B) shows the *OLA1* protein, with the secondary structure indicated by helices (pink), coils (brown), and strands (yellow). Panel (C) illustrates the tertiary structure of the OLA1 protein. Panel (D) presents the phylogenetic relationship of the human *OLA1* gene with other species at the nucleotide level.

Our sequence analyses unveiled that the coding DNA sequence (CDS) of the human *OLA1* gene spans 1191 base pairs, ultimately encoding a 396 amino acid protein, in agreement with findings in other species (Fig 2A). Structural analysis using PSIPRED (http://bioinf.cs.ucl.ac.uk/psipred/) for the 2D and 3D structures of the human OLA1 translated protein showed the presence of 13 strands, 16 helices, and 23 coils (Fig 2B and 2C).

Furthermore, phylogenetic analysis using MegAlign (Lasergene) based on both nucleotide and amino acid sequences indicated that the human *OLA1* gene shares a close evolutionary relationship with chimpanzees and monkeys, with the lowest similarity observed in zebrafish (89%) (Fig 2D). Percentage homology among different species and phylogeny is shown in S2A–S2C Fig. These findings provide valuable insights into the gene characteristics and evolutionary connections of *OLA1* among various species, including humans.

### Genomic organization of the human *OLA1* gene

For genomic characterization of the human *OLA1* gene, different fragments of the *OLA1* genomic sequence were amplified using different sets of primers (S1 Table) and confirmed based on their respective size with agarose gel analysis (S3A Fig). Different fragments of the *OLA1* gene were sequenced and assembled. The Open reading frame (ORF) of *OLA1* was determined by aligning with the human *OLA1* mRNA sequence and further validated using the online NCBI Splign program. This analysis revealed that the *OLA1* gene is composed of 11 exons, with the start codon located in exon 2 and the stop codon in exon 11 (S3B Fig). The length of each exon is also shown in S3B Fig and has been submitted to the NCBI gene data bank with an accession number ON073791.1. This information is of potential importance for determining the regulatory mechanisms that control the expression of the *OLA1* gene.

### Expression of the *OLA1* splice variant in human heart tissue

Several splice variants of the *OLA1* gene have been reported in both Ensembl human genome browser and the NCBI GenBank. To identify splice variants in the human heart, we designed several sets of primer targeting each variant, after aligning different OLA1 splice variant mRNA transcript reported in the Ensemble data bank. To find the splice variants, we amplified PCR product from reverse transcribed RNA isolated from human heart samples (S4 Fig). Our analysis revealed four distinct splice variants (202, 203, 205, and 209; S4 Fig) expressed in the human heart. Future studies are warranted to explore the specific roles of these splice variants in cancer and heart disease.

### Detection of mutations in the *OLA1* gene

In most cases (70–80%), familial DCM is inherited as an autosomal dominant disease, wherein a single copy of the altered gene is sufficient to precipitate the disorder. Previous studies have demonstrated a strong association between a locus on chromosome 2q31, designated as CAD1G, and familial DCM in humans. Notably, this locus encompasses the pivotal cytoskeletal muscle protein Titin [10]. Loss of function mutations in the [10, 16, 20]. Intriguingly, the *OLA1* gene mapped to the same locus on chromosome 2q31, in close proximity to the *TTN* gene. Alterations in the expression of *OLA1* have been identified in samples from patients with heart failure, as well as in the cardiac tissue of mice following ANGII administration [7, 8].

Studies in human cancer have shown that increased expression of *OLA1* correlated with swift disease progression and unfavorable prognosis [17]. A recent investigation in a breast cancer cell line demonstrated a point mutation (E168Q) in the *OLA1* gene, which impaired its ability to bind BRCA1 [3]. Additionally, research involving atherosclerosis patients has revealed several mutations in the *OLA1* gene, with five of them exhibiting a strong association with the development of atherosclerosis [18].

**Fig 3:**
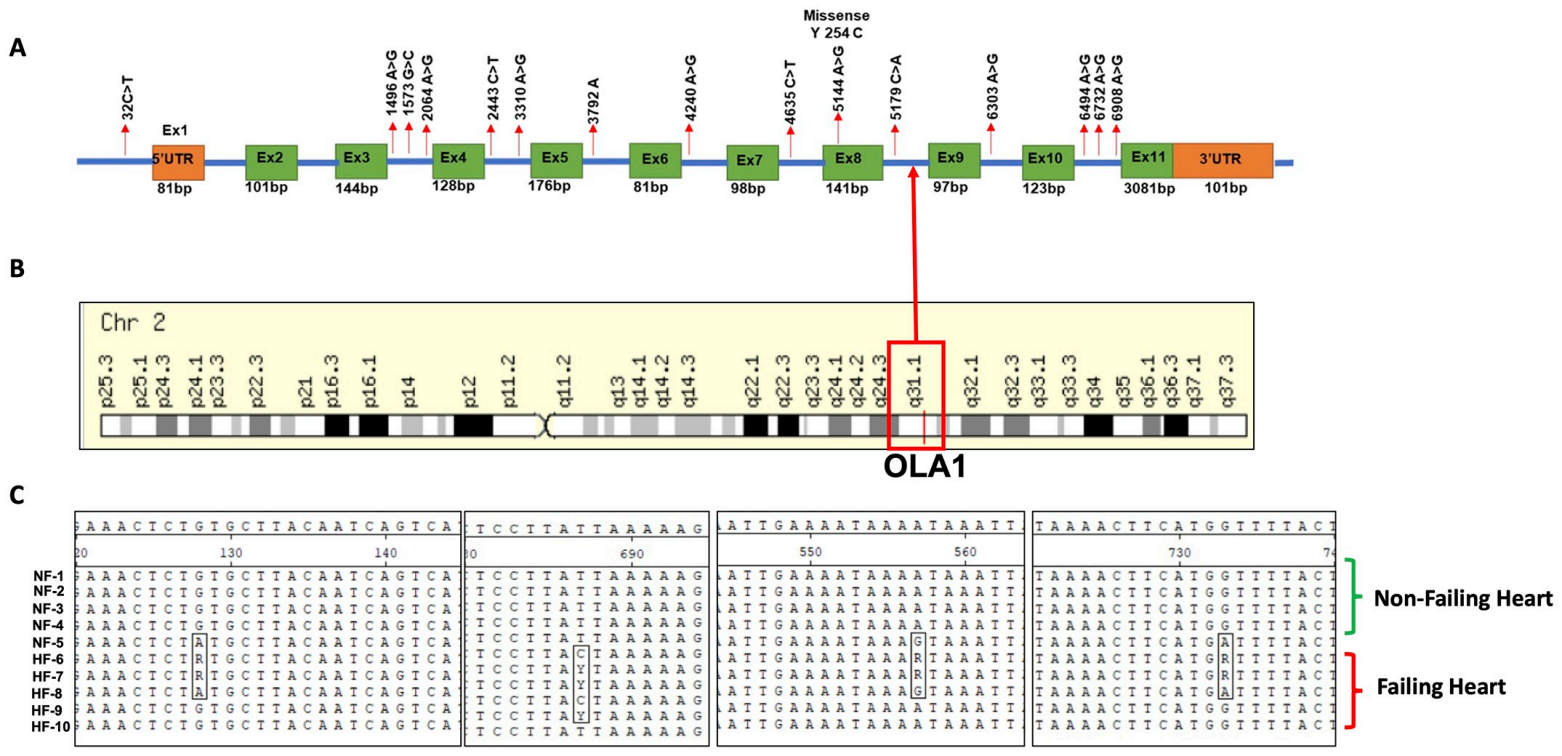
Mutations in the *OLA1* gene is found in both failing and non-failing cardiac patients. Panel (A) illustrates the distribution of mutations within exons and introns. Panel (B) shows the location of the *OLA1* gene on human chromosome 2, locus 2q31.1, which is associated with DCM in humans. Panel (C) displays a MegAlignment of nucleotide sequences, demonstrating that the *OLA1* mutations are interconnected and exhibit distinct patterns between failing (HF 6–10) and non-failing (NF1-6) cardiac patients.

To investigate the presence of mutations in the *OLA1* gene associated with heart disease, we performed amplification of the *OLA1* gene encompassing all exons as well as portions of the adjacent introns from DNA isolated from failing (n = 5) and non-failing (n = 5) human heart tissues. Through Sanger sequencing and subsequent sequence analysis, we identified 15 mutations, comprising two transversions, one substitution, one deletion, and 11 transitions in the *OLA1* gene (Fig 3, Table 1). The distribution of these single nucleotide polymorphisms (SNPs) among failing and non-failing heart patients is shown in S5 Fig. Remarkably, all the identified mutations were intronic, except for one non-synonymous mutation 5144A>G, resulting in 254Tyr>Cys, located in exon 8 of the *OLA1* gene. Furthermore, we conducted an in-depth analysis of the genotype and allelic frequencies of these identified SNPs in the human *OLA1* gene, as outlined in Table 1. The allele frequencies ranged from 0.1 to 0.35 among failing and non-failing heart patients.

**Table 1:** Mutations in OLA1 gene and its allelic and genotype frequency found in human heart failing and non-failing patients.

					Genotype Frequency			Allele Frequency	
Location	Region	Type	Non vs Synonymous	AA Change	AA	AB	BB	A	B
32 C>T	Promoter	Transition	-		0.6	0.2	0.2	0.70	0.30
1496 A>G	Intron3	Transition	-		0.5	0.3	0.2	0.65	0.35
1573 G>C	Intron3	Transversion	-		0.8	0.1	0.1	0.85	0.15
2064 A>G	Intron3	Transition	-		0.5	0.2	0.3	0.6	0.4
2443 c>t	Intron4				0.9	0.1	0	0.95	0.05
3310 A>G	Intron4	Transition	-		0.9	0.1	0	0.95	0.05
3792 A	Intron5	Substitution	-		0.8	0.2	0	0.9	0.1
4240 A>G	Intron6	Transition	-		0.1	0	0.9	0.1	0.9
4635 C>T	Intron7	Transition	-		0.2	0.3	0.6	0.35	0.75
5144 A>G	Exon8	Transition	Non-synon	254 Tyr > Cys	0.8	0.2	0	0.9	0.1
5791 C>A	Intron8	Transversion	-		0.2	0.2	0.6	0.3	0.7
6303 A>G	Intron9	Transition	-		0.2	0.2	0.6	0.3	0.7
6494 A>G	Intron10	Transition	-		0.8	0.2	0	0.9	0.1
6732 A>G	Intron10	Transition	-		0.6	0.2	0.2	0.7	0.3
6908 A>G	Intron10	Transition	-		0.2	0.2	0.6	0.3	0.7

* All positions respective to NCBI accession no. ON073791.1

### Association of *OLA1* mutations with heart failure

To investigate the potential association between *OLA1* mutations and heart failure, we compiled complete *OLA1* gene sequences from each patient and aligned them using MegAlign to identify genetic variations. Subsequently, we verified each variation by cross-referencing it with the respective chromatogram utilizing the Chromas Lite program. Intriguingly, we observed genetic variations in the DNA sequences of both failing and non-failing heart samples, displaying a notable correlation with failing hearts. The genotype and allele frequency patterns exhibited significant distinctions between failing and non-failing patients (Table 1), and these SNPs exhibited interlinkage to each other, as demonstrated in the MegAlign (Fig 3C). Further analysis showed that the majority of these SNPs were located within the intronic regions, displaying mutual correlations, and implying their interdependence (Fig 3C).

**Fig 4:**
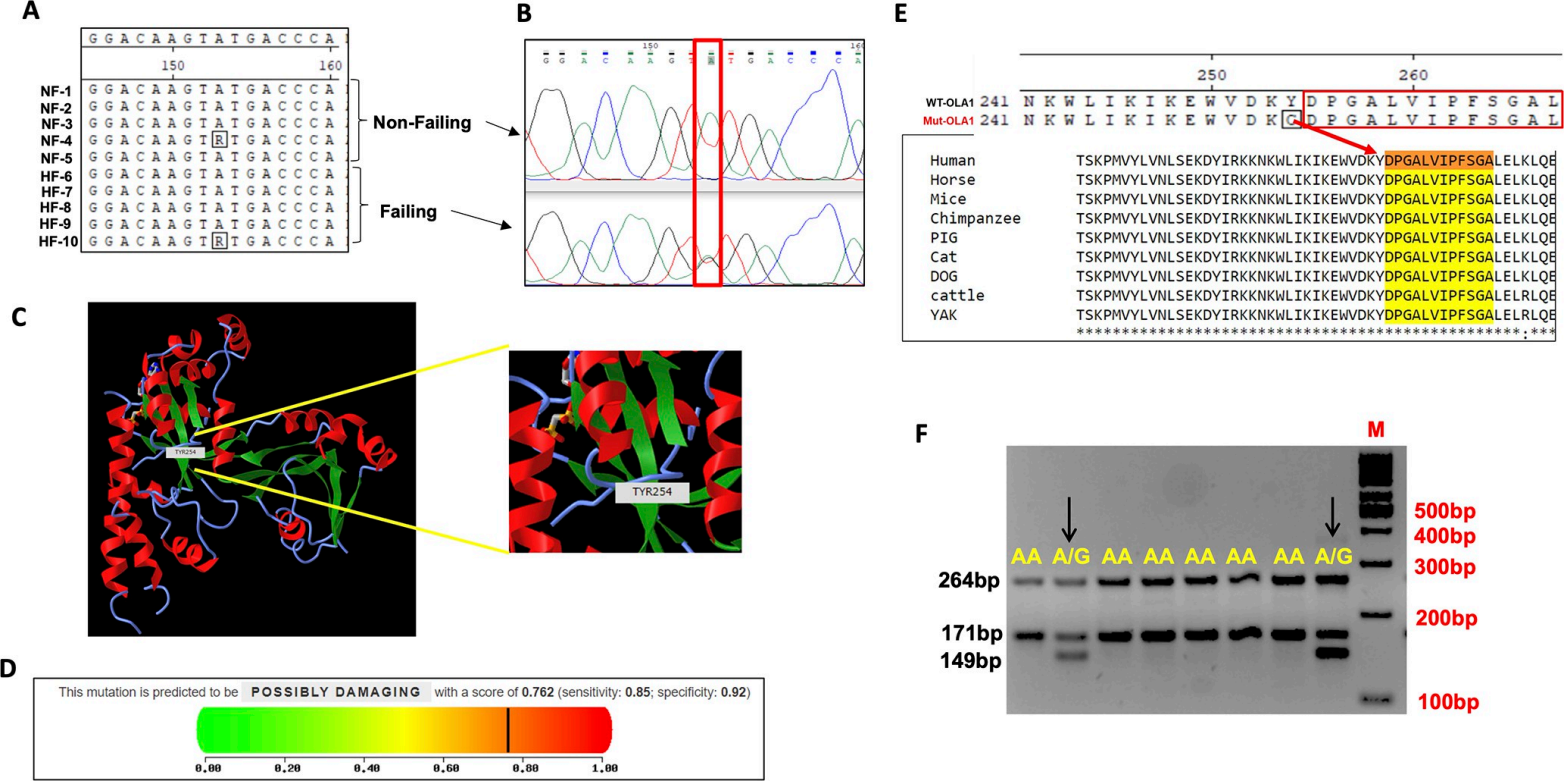
Non-synonymous mutation (254Tyr>Cys) in exon 8 of the *OLA1* gene. Panel (A) provides a MegAlignment, demonstrating a heterozygous (R, A>G) change in exon 8 of the *OLA1* gene. Panel (B) displays a chromatogram showing the shift from a homozygous AA to a heterozygous A>G peak. Panel (C) presents the Phyphen-2 analysis of the 254 Tyr>Cys mutation, indicating its detrimental impact on protein function. Finally, panel (D) shows the three-dimensional structure of the OLA1 mutant protein. (E) Depicts the conservation of the mutation in OLA1 (254Tyr>Cys) across different species. (F) Demonstrates the development of a PCR-based assay for screening the *OLA1* mutation (5144A>G).

Additionally, we identified a heterozygous exonic mutation (5144A>G, resulting in 254Tyr>Cys) in exon 8 (Fig 4A–4C), which has also been reported in the NCBI dbSNP data bank under ID (rs11558990). We further assessed the potential impact of this mutation using various online servers. The Predictor of Human Deleterious Single Nucleotide Polymorphisms (PhD-SNP) analysis indicated that this *OLA1* mutation (254Tyr>Cys) is deleterious in nature [19]. Similar predictions were also obtained from PhyPhen2 (0.89) [24] and SIFT (0.78) analyses [20] (Fig 4D).

Interestingly, we found a distinct pattern of *OLA1* mutations among failing and non-failing heart patients, with certain SNPs exhibiting associations with each other (Fig 3C) resulting in different haplotypes among these two conditions (Table 2). Additionally, the presence of several mutations in the intronic regions of *OLA1* suggests a potential RNA splicing defect contributing to OLA1 misregulation in heart disease, and warrants further exploration.

**Table 2:** Haplotypes observed in the human *OLA1* gene.

	Sample	32	1496	1573	2064	2443	3310	3792	4240	4635	5144	5791	6303	6494	6732	6908
**Non Failing**	AOC-6	T	G	G	G	Y	A	A	G	C	A	A	A	G	G	A
	AOC-11	Y	A	S	A	T	A		G	T	A	C	G	G	A	G
	AOC-13	Y	R	G	R	T	R		G	T	R	C	G	R	A	G
	AOC-18	Y	A	G	A	T	A		G	T	A	C	G	G	A	G
	AOC-20	C	A	C	A	T	A		G	T	A	C	G	G	A	G
**Failing**	**HTX-40**	**T**	**R**	**G**	**R**	**T**	**A**		**G**	**Y**	**A**	**M**	**R**	**G**	**R**	**R**
	**HTX-49**	**T**	**R**	**G**	**R**	**T**	**A**	**A**	**G**	**Y**	**A**	**M**	**R**	**G**	**R**	**R**
	HTX-58	T	G	G	G	T	A	A	G	C	A	A	A	G	G	A
	HTX-70	Y	A	G	A	T	A		G	T	A	C	G	G	A	G
	HTX-60	Y	A	G	A	T	A		R	Y	R	C	G	R	A	G

*Heterozygote code represent R-A/G, S-G/C, M-A/C, Y-C/T

### Development of a PCR-based screening test for the *OLA1* point mutation (254Tyr>Cys)

The non-synonymous mutation 5144A>G was identified in the *OLA1* gene in both failing and non-failing heart patients. This mutation has also been reported in the SNP data bank and among cancer patients. Crystallographic analysis of the mutant OLA1 protein revealed an altered structural conformation compared to the wild-type OLA1 [1]. In light of these findings, coupled with the proximity of SNP 5144A>G (254Tyr>Cys) to the GTPase domain of the OLA1 and its highly conserved sequence across species (Fig 4E), we devised a streamlined, cost-effective, and expeditious screening protocol for the detection of this point mutation.

To accomplish this, we developed a Tetra-ARMS PCR screening assay utilizing four distinct primer sets. The two outer primers (forward and reverse) were common for both genotypes, amplifying a 264 bp fragment. Conversely, each of the inner primer sets was allele-specific. The inner forward primer designed for the G allele generated a 149 bp fragment, while the inner reverse primer tailored for the A allele produced a 171 bp fragment (Fig 4F). This screening protocol underwent further validation using known genotypes to ascertain its precision. As anticipated, the AA genotype exhibited two bands (264bp and 171bp), while the heterozygous A/G genotype displayed three bands (264bp, 171bp, and 149 bp) (Fig 4F). To further corroborate the existence of this mutation in the human *OLA1* gene, we examined a large dataset in the NCBI SNPs variant dataset. The exonic non-synonymous mutation 5144A>G (254Thr>Cys) was also observed in these datasets.

## Discussion

In the present study, we found that both mRNA and protein expression of *OLA1* were significantly downregulated in failing human heart tissue compared to non-failing tissue, underscoring the importance of OLA1 in cardiac physiology. This is consistent with a previous study where mass spectrometric analysis also showed a downregulation of OLA1 expression in a failing human heart [7]. Furthermore, in an Angiotensin II (AngII)-induced cardiac stress mice model, the expression of OLA1 was altered, which led to the phosphorylation of the GSK-3β/β-catenin pathway [8].

OLA1 belongs to a GTPase family of proteins and has both GTPase and ATPase activities. The elevated expression of OLA1 in cancer cells has been correlated with poor survival and clinical outcomes [1, 17]. Increased expression of OLA1 in cancer cells appears to enhance their survival by interacting with BRCA1 and BRCA1-associated RING domain proteins (BARD1) [3]. Moreover, OLA1 appears to interact with Hsp70 to stabilize SOD2 in pulmonary smooth muscle cells in mice. Depletion of OLA1 leads to SOD2 deficiency, resulting in increased expression of the anti-apoptotic gene, X-linked inhibitor of apoptosis (XIAP), and consequently, an elevated proliferation of pulmonary smooth muscle cells [4, 5]. A knockout of OLA1 in mice (null mutants) resulted in stunted growth, delayed development, and the birth of litters with immature organs, ultimately leading to perinatal lethality, highlighting the essential role of OLA1 in growth and survival [4]. Interestingly, this global *OLA1* knockout also led to an increase in heart weight relative to body weight, indicating the potential significance of OLA1 in cardiac pathophysiology [4].

Given the importance of OLA1 in heart diseases, we characterized *OLA1* at both mRNA and genomic levels and compared it with other species. Our findings demonstrated that the *OLA1* gene exhibits maximum homology with other species, suggesting that the OLA1 function is evolutionarily conserved and has a crucial role in cellular physiology across species. This conservation of homology across species is similar to the conservation of homology across species of another gene called *Tbx5*. Tbx5 also plays a critical role in heart development [21, 22].

The significance of mRNA splicing in post-transcriptional RNA modifications, resulting in a single gene encoding multiple isoforms of proteins to regulate biological processes differentially, has been well documented. This is also the case in heart development, where multiple isoforms of sarcomere genes have been associated with heart failure [23]. Notably, the *TTN* gene, encoding a major sarcomere protein, produces multiple isoforms through alternative splicing, and mutations in *TTN* contribute to the development of DCM in humans [23–25]. The *OLA1* gene maps close to *TTN* (chromosome 2, locus 2q31). Our study also identified multiple mRNA splice isoforms of *OLA1* in human cardiac tissue, warranting further validation of their function in cardiac physiology.

DCM is characterized by a progressive dilation of the left ventricle and systolic dysfunction, ultimately leading to heart failure. It is the most genetically heterogeneous form of cardiomyopathy, with multiple forms of the disease often linked to the same gene [9]. This underscores the importance of genomic context in the pathophysiology of disease-associated variants. Similarly, mutations in over 40 genes encoding the components of the sarcomere, cytoskeleton, or nuclear lamina, are implicated in DCM in humans [9, 10]. Among these, truncation or point mutations in the *TTN* gene is associated with familial DCM [10]. These lesions in the *TTN* gene appear to account for up to 25% of DCM cases in humans [10, 16]. Intriguingly, the *OLA1* gene is also mapped to the same locus (2q31 of chromosome 2) as *TTN*. Recent studies have indicated that mutations in *OLA1* are associated with poor survival and an increased risk of developing human atherosclerotic diseases [17, 18, 26].

The effects of *OLA1* mutations on heart failure in human patients are yet to be extensively studied. In this investigation, we screened for mutations in the *OLA1* gene and explored their association with heart failure in humans. Our study uncovered 15 mutations in the *OLA1* gene among both failing and non-failing heart patients. These mutations were primarily located within the intronic regions, except for a nonsynonymous 5144A>G change, resulting in an amino acid substitution from Tyr to Cys at position 254. This mutation, also reported in the dbSNP database (NCBI) under SNP ID rs11558990, has been strongly correlated with rapid disease progression and poor survival in cancer patients. OLA1 maps to chromosome 2 (locus 2q31.1), near Titin (TTN), which is associated with familial dilated cardiomyopathy (DCM) in humans. Mutations in this locus are linked to heart diseases and are designated as the dilated cardiomyopathy locus (CMD1G) [27]. Recent studies have also reported mutations in the OLA1 gene associated with the incidence of atherosclerosis in humans [18]. A point mutation (E168Q) in *the OLA1* gene is linked to apoptosis, preventing the formation of a complex with BRCA1 and BARD1, and is associated with poor outcomes in breast cancer [3]. Apoptosis is a critical cellular process in regulating cardiomyocyte death during heart failure. Our identified mutation, 254Tyr>Cys, might also play an important role in regulating cardiomyocyte death and is critical to cardiovascular function during stress conditions such as heart failure, which warrants further investigation. Our study also identified the same mutation in the *OLA1* gene among patients with heart disease. This point mutation could have a strong negative impact on heart function. For example, another point mutation in *OLA1* (E168Q) prevents it from forming a complex with BRCA1 and BARD1 and is associated with poor outcomes in breast cancer [3]. Given the likely importance of the 254Tyr>Cys point mutation in heart disease, we have developed a rapid, reliable, and cost-effective genotyping protocol using the Tetra-ARMS PCR method [11–13, 28]. Using isolated DNA, this protocol helps test genetic missense mutations in the OLA1 (5144A>G, 254Tyr>Cys) gene. Because this is a germline, inherited mutation, not somatic, it is expected to be present in all cells in the body. Given that OLA1 mutation(s) is also dominant or semi-dominant, a DNA-based test, as the one described here, of any sample cells from an individual will detect the mutation in heterozygous condition. In this paper, we tested only DNA from heart samples, but the test could be applied to blood samples as well. We believe this PCR-based assay will be highly useful to noninvasively screen for risks of developing heart disease due to a mutation in *OLA1* using easily accessible cells or tissues, such as blood cells. It will also be useful for screening somatic mutations in breast and other cancers, highlighting the broad application of this test. In summary, our findings shed light on the potential role of OLA1 in cardiac physiology and underscore the significance of *OLA1* mutations in heart failure.

### Study limitation

To establish a robust association between mutations in the OLA1 gene and heart disease, examining samples from a larger sample size is necessary. Also, in the present study, we did not consider other confounding factors and instead focused directly on patients with failing versus non-failing hearts, and mainly the heart tissue. Including confounders is important but would require a large-scale study involving many patients, which was beyond the scope of this work. Future studies with larger sample sizes will have to consider these confounding factors to provide a more comprehensive understanding of the relationship between OLA1 mutations and heart disease.

## Supporting information

S1 Fig Expression and localization of *OLA1* in mouse tissue and human cardiomyocyte (AC16) cell line. (A) Immunoblot showing the expression of the OLA1 and GAPDH proteins in various mouse tissues. (B) Representative graph after normalization of OLA1 with GAPDH in different tissues is shown (n = 3) **(C)** Cytoplasmic and Nuclear localization of OLA1, GAPDH (Cytoplasmic Marker) and Lamin B1 (Nuclear marker) in human cardiomyocyte (AC16) cells.
(TIFF)

S2 Fig Percentage homology and phylogenetic analysis of the human OLA1 gene with different species at nucleotide and amino acid levels. (A) Percentage identity of the human *OLA1* gene with other species at the nucleotide level. (B) Percentage identity of the human OLA1 protein with other species at the amino acid level. (C) Phylogenetic analysis shows that the *OLA1* gene is evolutionarily conserved among different species.
(TIFF)

S3 Fig Agarose Gel electrophoresis showing PCR amplification of human *OLA1* gene from isolated DNA from human heart (A) Agarose gel showing different fragments of *OLA1* gene amplification covering all exons along with adjacent introns from isolated DNA (B) Genomic organization of the *OLA1* gene. Ex (Exon), UTR (Untranslated Region), CDS (Coding DNA sequence), bp (Base pair).
(TIFF)

S4 Fig Semi quantitative amplification of different transcript variants of the *OLA1* gene expressed in the human heart. (TIFF)

S5 Fig Allelic distribution of SNPs (A) Allelic distribution of point mutations found in human *OLA1* gene among failing and non-failing heart patients. (TIFF)

S1 Table List of primers. (PDF)

S1 Raw images (PDF)
