# Supplementary figures and images for: Identification and development of Tetra-ARMS PCR-based screening test for a genetic variant of OLA1 (Tyr254Cys) in the human failing heart
Source: PLoS One. 2024 Jun 18;19(6):e0293105. doi: 10.1371/journal.pone.0293105 (PMC11185490; doi:10.1371/journal.pone.0293105)

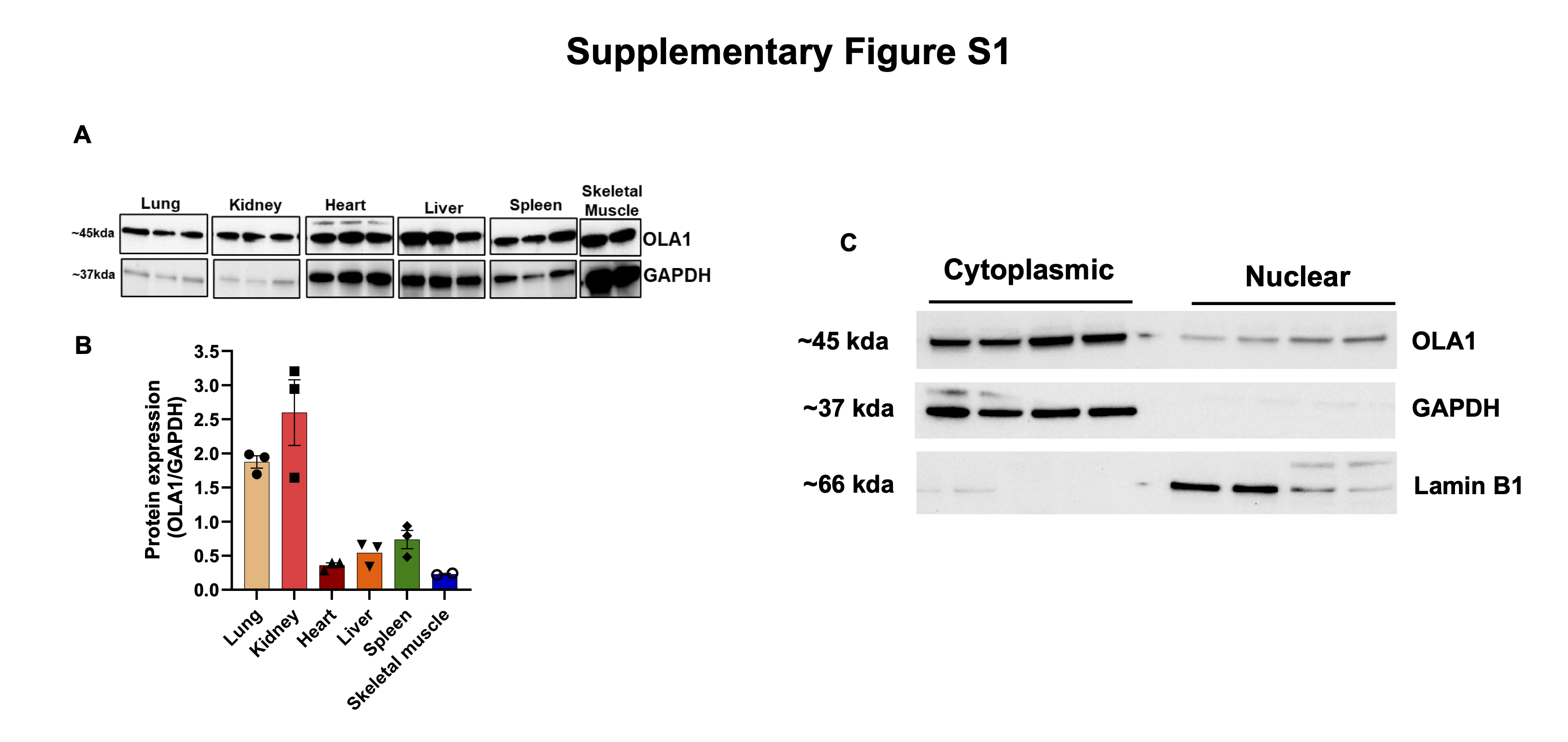

Supplement: S1 Fig — (A) Immunoblot showing the expression of the OLA1 and GAPDH proteins in various mouse tissues. (B) Representative graph after normalization of OLA1 with GAPDH in different tissues is shown (n = 3) (C) Cytoplasmic and Nuclear localization of OLA1, GAPDH (Cytoplasmic Marker) and Lamin B1 (Nuclear marker) in human cardiomyocyte (AC16) cells. (TIFF) [file pone.0293105.s001.tiff]

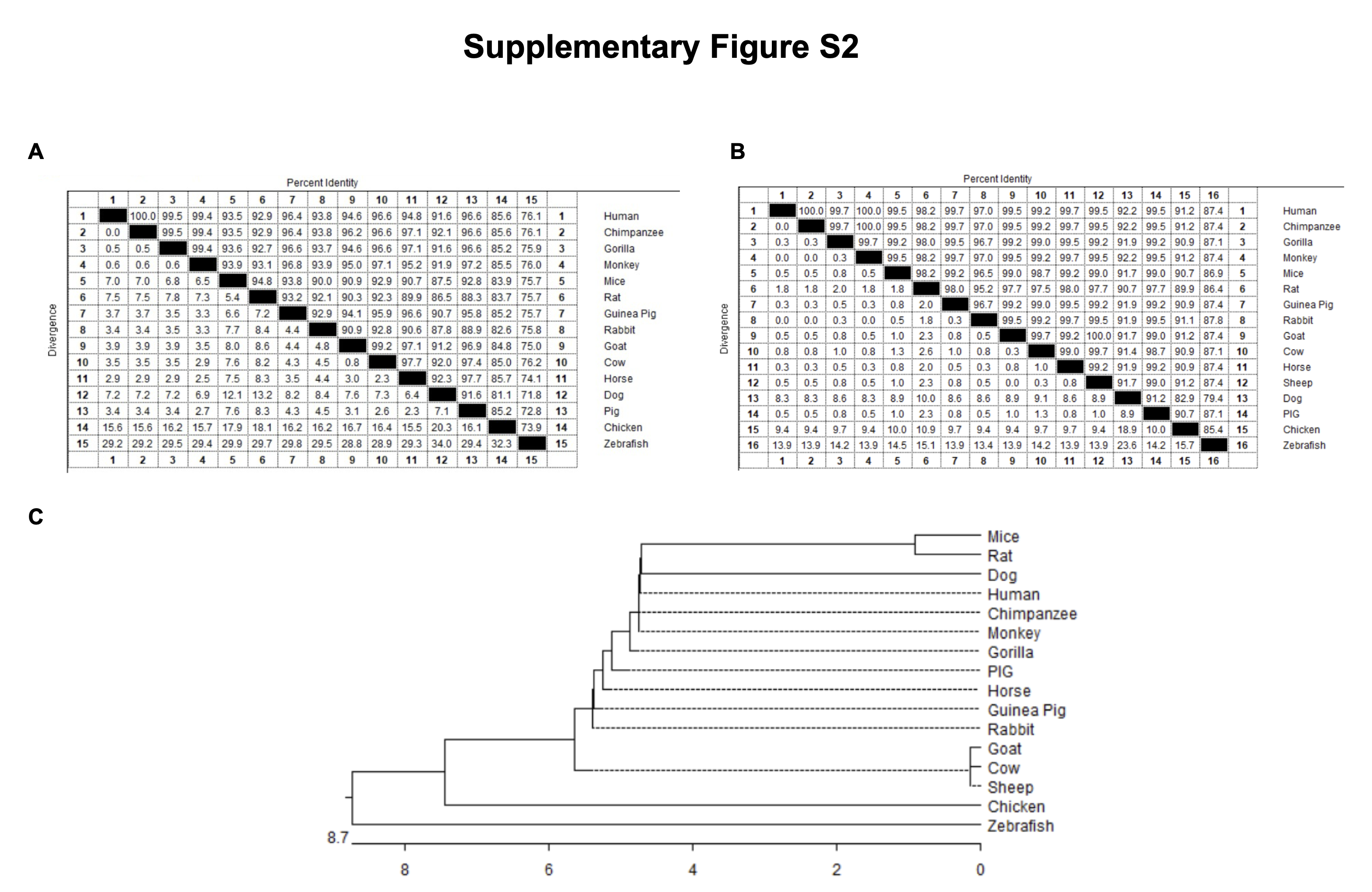

Supplement: S2 Fig — (A) Percentage identity of the human OLA1 gene with other species at the nucleotide level. (B) Percentage identity of the human OLA1 protein with other species at the amino acid level. (C) Phylogenetic analysis shows that the OLA1 gene is evolutionarily conserved among different species. (TIFF) [file pone.0293105.s002.tiff]

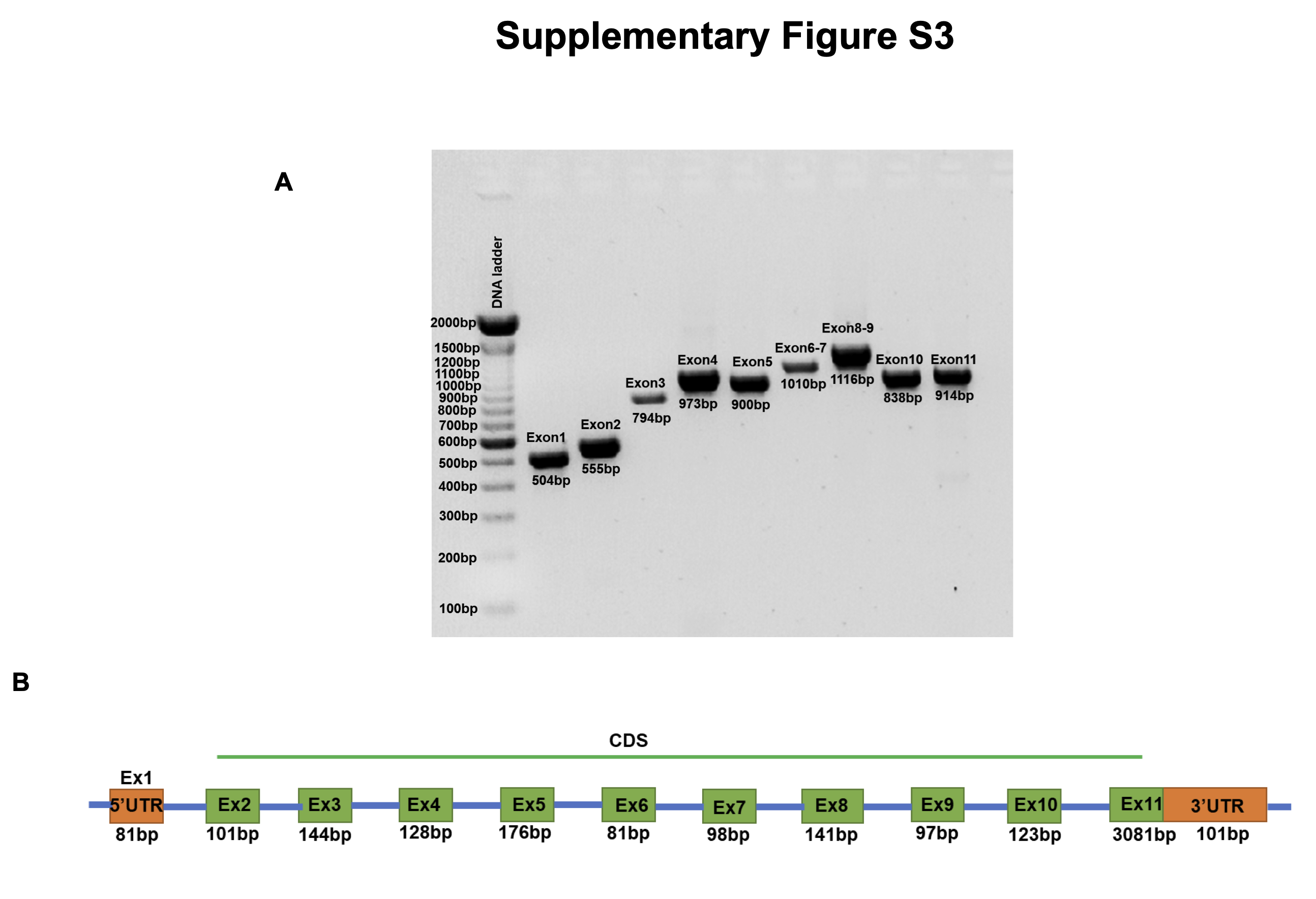

Supplement: S3 Fig — Ex (Exon), UTR (Untranslated Region), CDS (Coding DNA sequence), bp (Base pair). (TIFF) [file pone.0293105.s003.tiff]

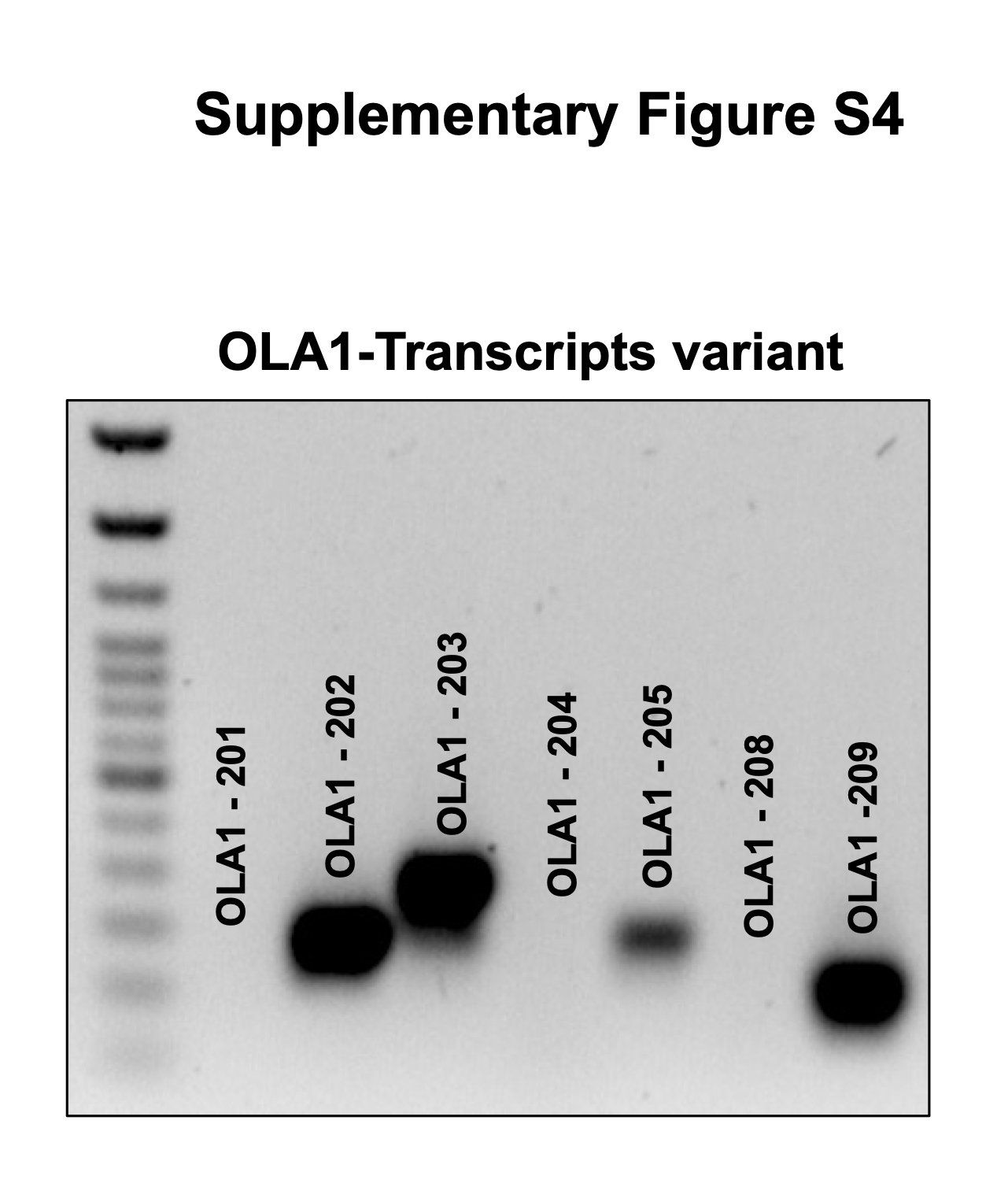

Supplement: S4 Fig — (TIFF) [file pone.0293105.s004.tiff]

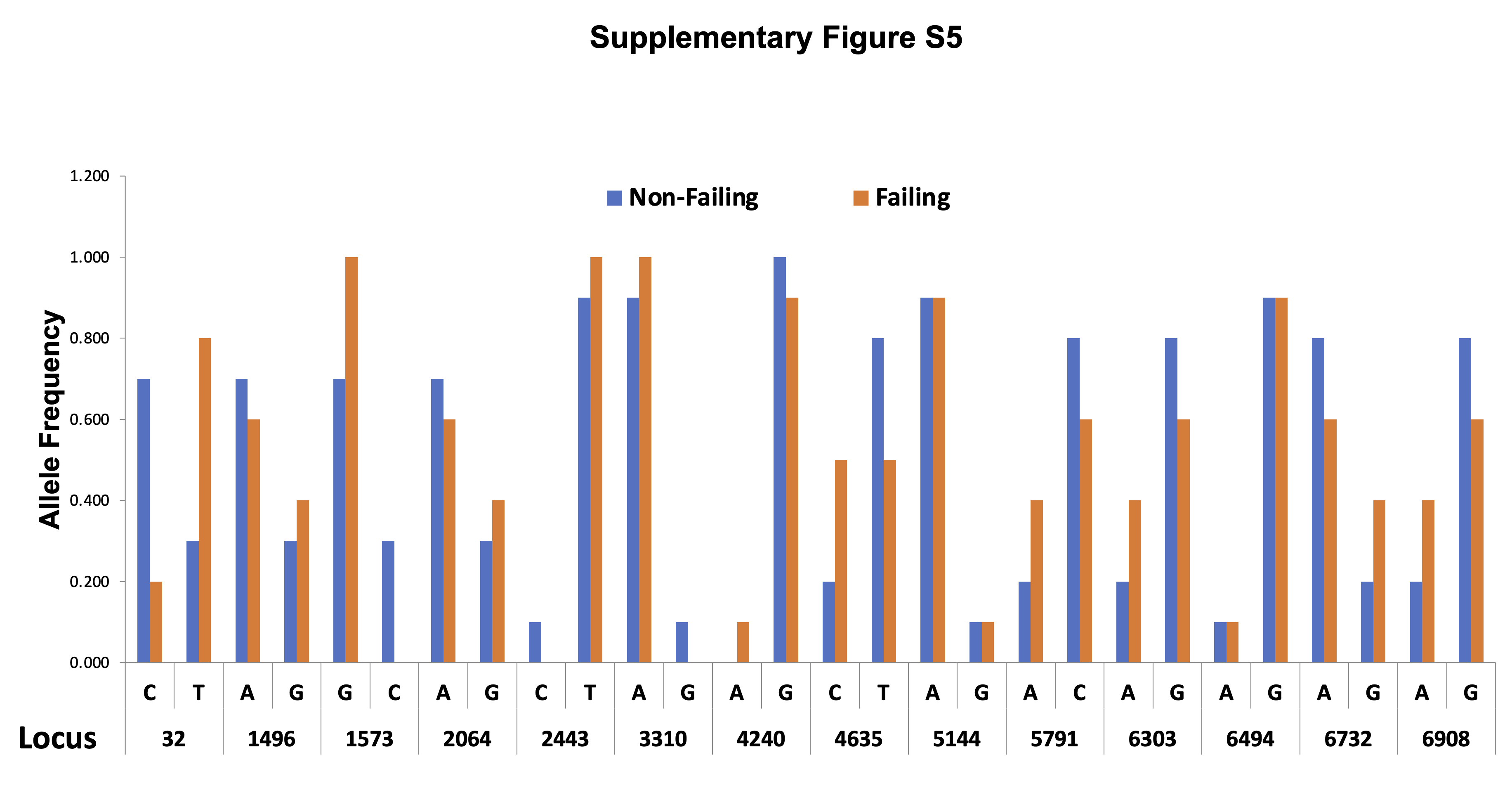

Supplement: S5 Fig — (TIFF) [file pone.0293105.s005.tiff]
