# Supplementary material for: Identification and development of Tetra-ARMS PCR-based screening test for a genetic variant of OLA1 (Tyr254Cys) in the human failing heart
Source: PLoS One. 2024 Jun 18;19(6):e0293105. doi: 10.1371/journal.pone.0293105 (PMC11185490; doi:10.1371/journal.pone.0293105)
Supplement: S1 Table — (PDF) [file pone.0293105.s006.pdf]

Supplementary Table-1: List of Primers used in the study

PRIMERS				
**NAME**	**Forward**	**Reverse**	**Annealing Temp**	**Size (bp)**
**OLA1-EXON1**	GCCGAAGCGGAAGACTAGAG	CATCCACACATCCCCAAGCC	58°C	504
**OLA1-EXON2**	CTGGGACTTCTCAGCAATGACT	GCATGGCCTCTGGTAGACAG	58°C	555
**OLA1-EXON3**	ATGAGCTGCCGCTTTACTGT	ACCCTTCTACACCCTTCTCCA	58°C	794
**OLA1-EXON4**	CACCGCTTTTGAGTGTGTGTG	CAGAGCCCAAACTAAGCCAC	58°C	974
**OLA1-EXON5**	CCCTCAAATCAGGCCGATGA	CCCTCAAATCAGGCCGATGA	58°C	900
**OLA1-EXON-6-7**	GTGACCTAGCCCCCTTTCATT	GCTACAACCCCAGGTGGGTA	58°C	1010
**OLA1-EXON-8-9**	TGCTGCCTGTTAATGCCTTG	CCGCCACCATGACAAACTTC	58°C	1166
**OLA1-EXON-10**	AGGAAAAACAAAGCCACGCT	ACCTGCCCTCTAGTGTTGAGA	58°C	838
**OLA1-EXON-11**	TGACTTGCTGGATGTTTGCAG	CGTATCGCATTCAGCACACC	58°C	914
**OLA1-ORF**	ATGCCCCCTAAAAAGGGAGGTG	TTATTTCTTCTTCGGTTGTTGAGGTG	58°C	1191
**OLA1-ORF**	ATGCCCCCTAAAAAGGGAGGTG	TTATTTCTTCTTCGGTTGTTGAGGTG	58°C	1191
**OLA1-201**	AGCACGATGCCCCCTAAAAA	GGCCAGCAATATCCACCACAT	58°C	292
**OLA1-202/RT**	GCCTGCGTTCTCTCCTCCTTC	GGCCAGCAATATCCACCACAT	58°C	283
**OLA1-203**	CTGGTTGGAGGTCACCAGT	AGTTCCAAGGCCCCACTAAA	58°C	295
**OLA1-204**	CCCAGTACCTGATGCCCCC	GGCCAGCAATATCCACCACAT	58°C	297
**OLA1-205**	CTGGGACTTCTCAGCAATGACT	CCTCCCTTTTTAGGGGGCAT	58°C	137
**OLA1-208**	TGTGCAAAGTAAAATCCTGGGT	GACCAAAGCACCTGGGTCAT	58°C	224
**OLA1-209**	GCTGCACAGATACTACCCAGT	CTTCGGTTGTTGAGGTGTGT	58°C	388
**GAPDH RT**	AGGTCGGTGTGAACGGATTTG	TGTAGACCATGTAGTTGAGGTCA	60°C	120
**GENO-1**	GATAAAAATTAAAGAGTGGGTGGACAATTG	Forward Inner primer (G allele)	58°C	149
**GENO-2**	AGGAATGACCAAAGCACCTGGGTAAT	Reverse inner primer (A allele)	58°C	171
**GENO-3**	GCATCATAAAACCGTGCAATTCTTATG	Forward outer primer	58°C	264
**GENO-4**	GCTAATTAACCTTTGTGTCATGTTCGCT	Reverse outer primer	58°C	264
