# Supplementary material for: Identification and development of Tetra-ARMS PCR-based screening test for a genetic variant of OLA1 (Tyr254Cys) in the human failing heart
Source: PLoS One. 2024 Jun 18;19(6):e0293105. doi: 10.1371/journal.pone.0293105 (PMC11185490; doi:10.1371/journal.pone.0293105)

Figure-1B

Cropped blots

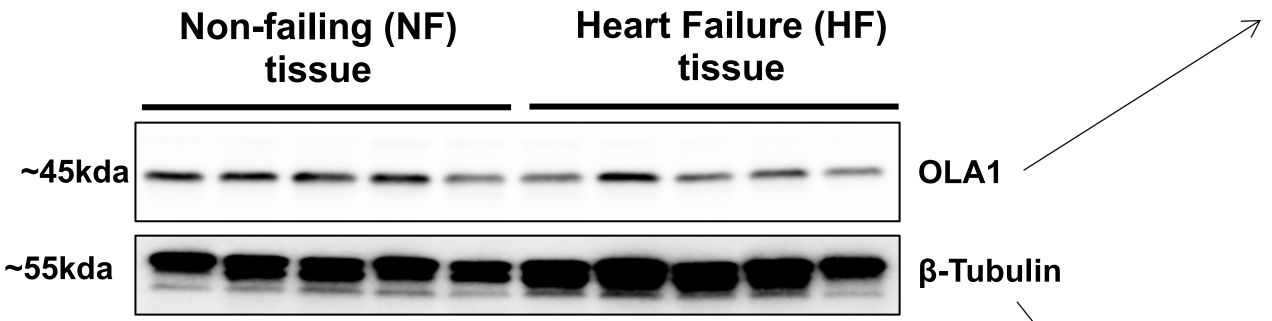

Uncropped blots

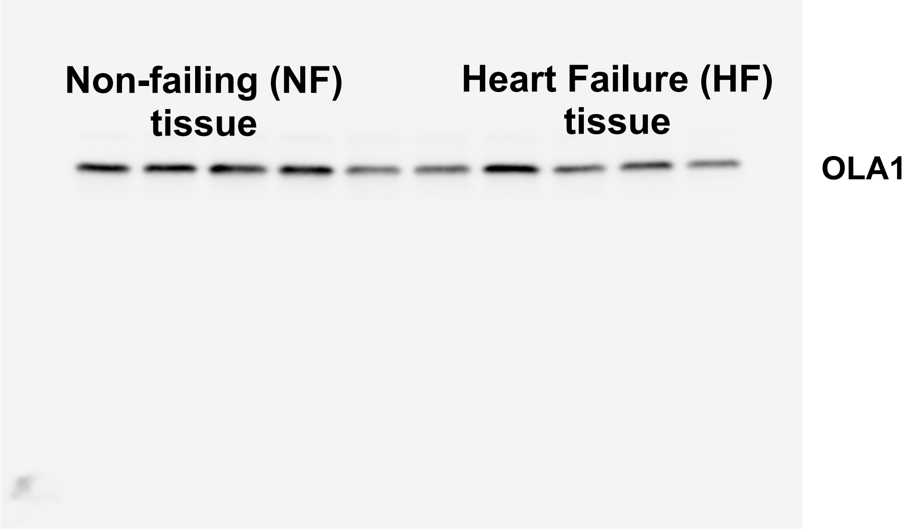

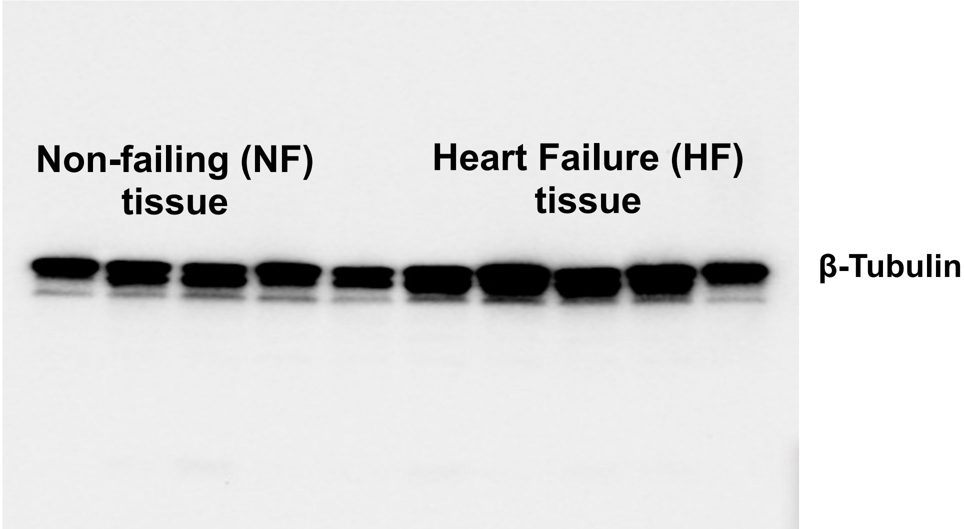

Figure-4F

Cropped blots

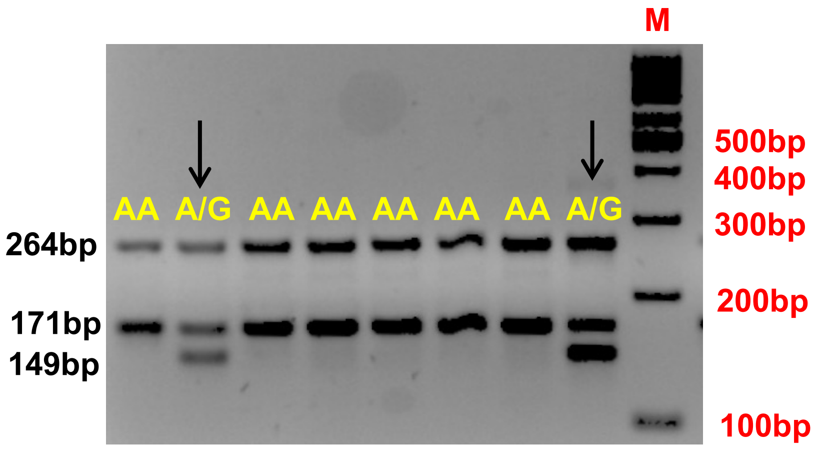

Uncropped blots

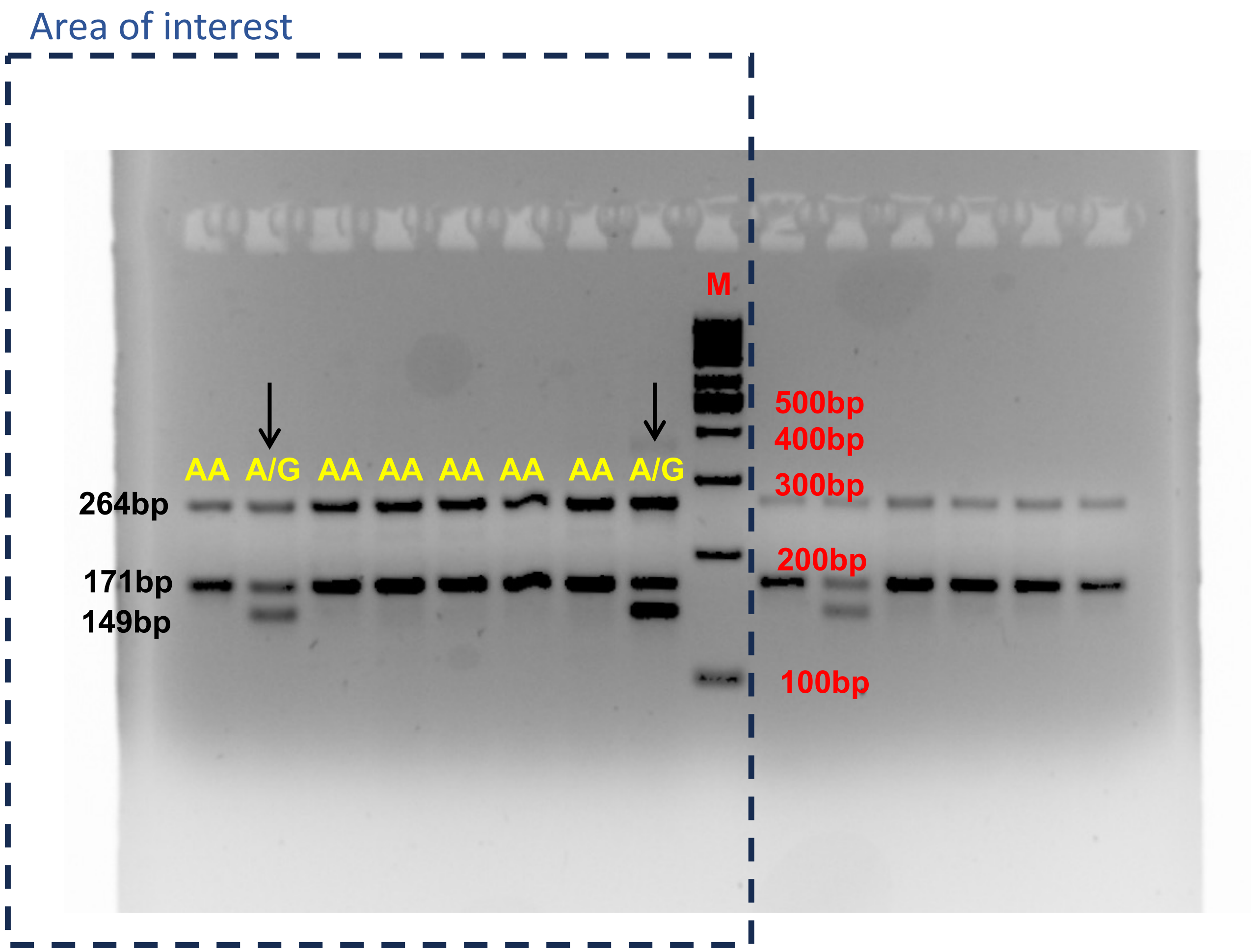

Supplementary Figure S1A

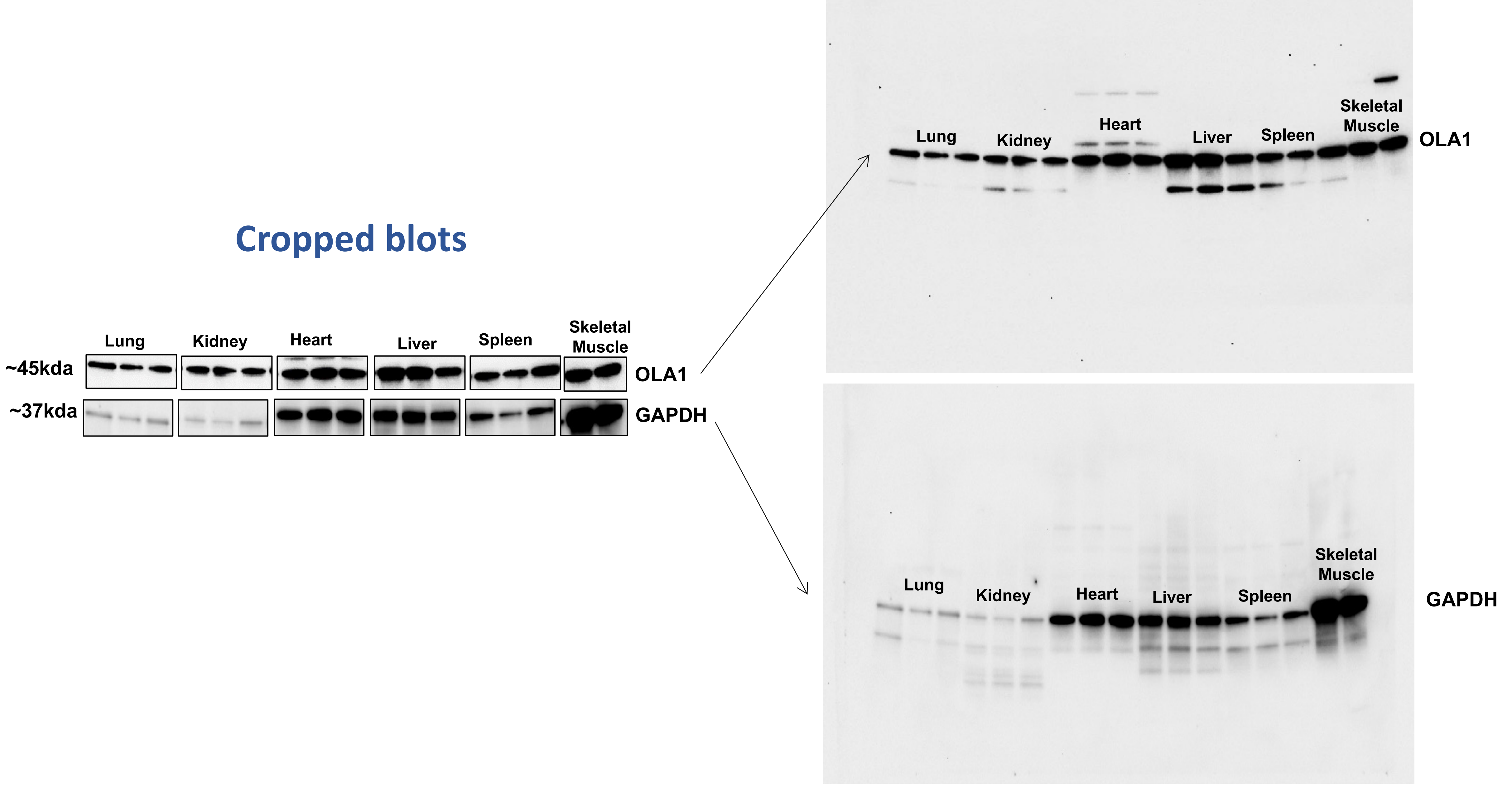

Supplementary Figure 1C

Cropped blots

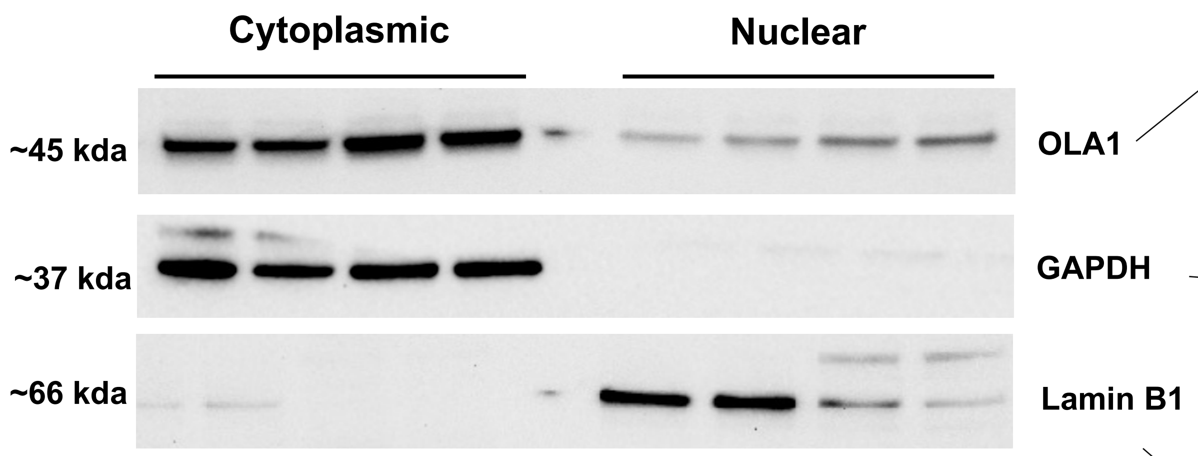

Uncropped blots

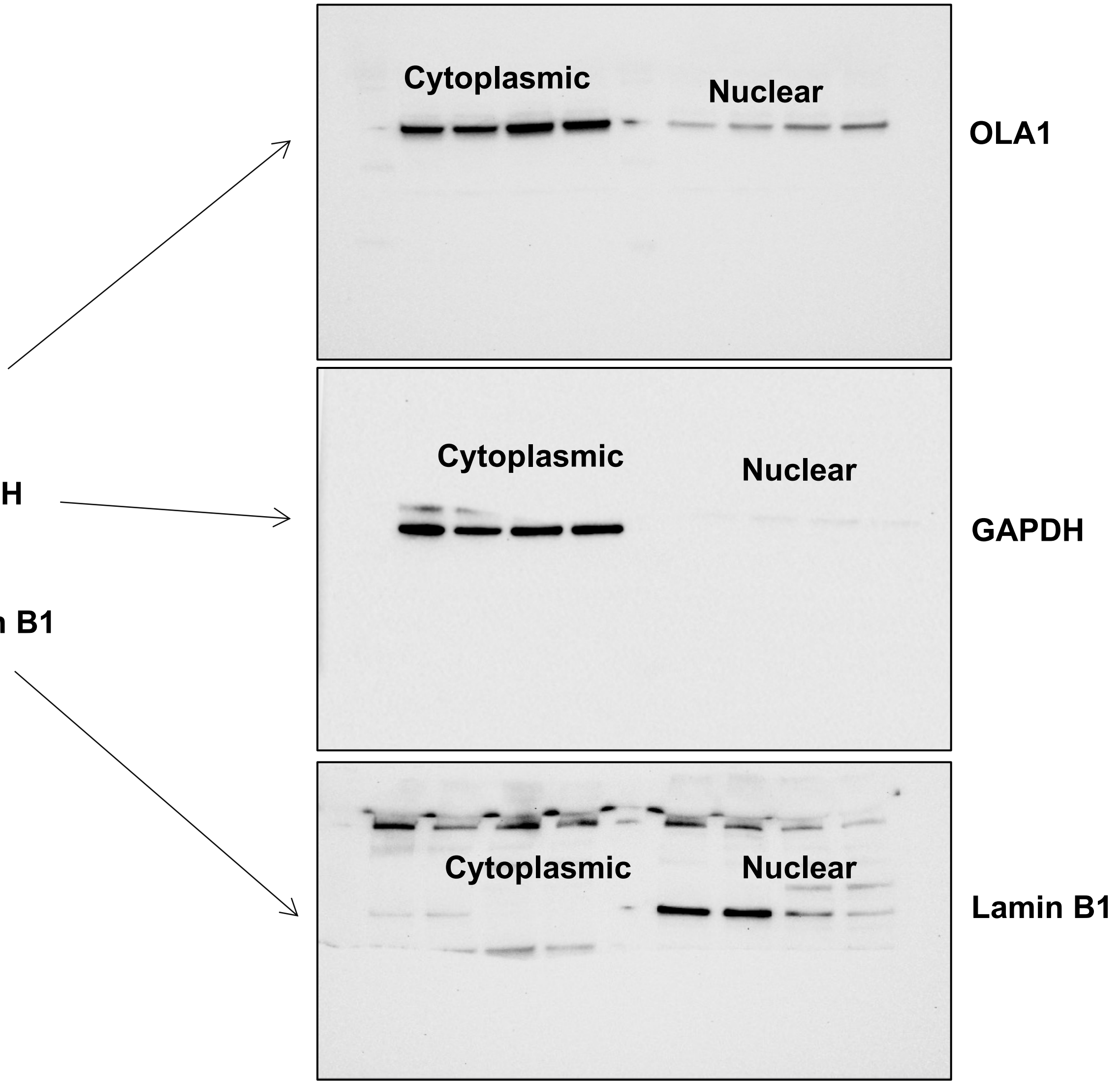

Supplementary Figure S3

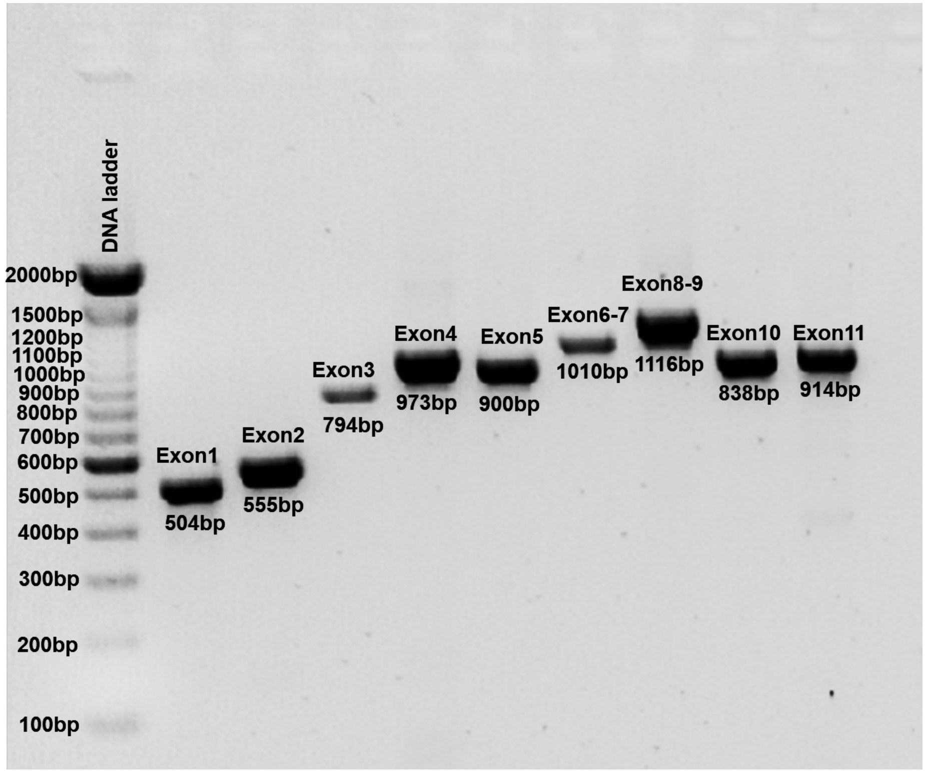

Supplementary Figure S4. Semiquantative Amplification of different transcript variants of the *OLA1* gene expressed in the human heart.

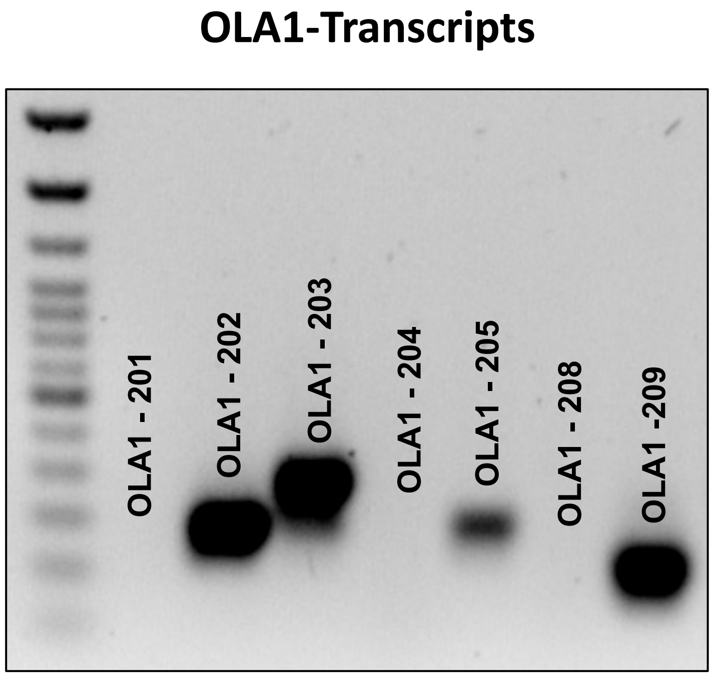

Supplement: S1 Raw images — (PDF) [file pone.0293105.s007.pdf]
